# Implementation of antibiotic stewardship programmes in paediatric patients in regional referral hospitals in Tanzania: experience from prescribers and dispensers

**DOI:** 10.1093/jacamr/dlac118

**Published:** 2022-11-23

**Authors:** Lilian Nkinda, Dorkasi L Mwakawanga, Upendo O Kibwana, Wigilya P Mikomangwa, David T Myemba, Nathanael Sirili, Rodgers Mwakalukwa, Manase Kilonzi, Godfrey Sambayi, Betty A Maganda, Belinda J Njiro, Harrieth P Ndumwa, Ritah Mutagonda, Alphonce I Marealle, Fatuma F Felix, Hamu J Mlyuka, Gerald Makuka, Samson W Kubigwa, Peter P Kunambi, Rashid Mfaume, Arapha Bashir Nshau, George M Bwire, Robert Scherpbier, Elevanie Nyankesha

**Affiliations:** School of Medicine, Muhimbili University of Health and Allied Sciences, PO Box 65001, Dar es Salaam, Tanzania; School of Nursing, Muhimbili University of Health and Allied Sciences, PO Box 65001, Dar es Salaam, Tanzania; School of Medicine, Muhimbili University of Health and Allied Sciences, PO Box 65001, Dar es Salaam, Tanzania; School of Pharmacy, Muhimbili University of Health and Allied Sciences, PO Box 65013, Dar es Salaam, Tanzania; School of Pharmacy, Muhimbili University of Health and Allied Sciences, PO Box 65013, Dar es Salaam, Tanzania; School of Public Health and Social Sciences, Muhimbili University of Health and Allied Sciences, PO Box 65015, Dar es Salaam, Tanzania; School of Pharmacy, Muhimbili University of Health and Allied Sciences, PO Box 65013, Dar es Salaam, Tanzania; School of Pharmacy, Muhimbili University of Health and Allied Sciences, PO Box 65013, Dar es Salaam, Tanzania; School of Pharmacy, Muhimbili University of Health and Allied Sciences, PO Box 65013, Dar es Salaam, Tanzania; School of Pharmacy, Muhimbili University of Health and Allied Sciences, PO Box 65013, Dar es Salaam, Tanzania; School of Medicine, Muhimbili University of Health and Allied Sciences, PO Box 65001, Dar es Salaam, Tanzania; School of Medicine, Muhimbili University of Health and Allied Sciences, PO Box 65001, Dar es Salaam, Tanzania; School of Pharmacy, Muhimbili University of Health and Allied Sciences, PO Box 65013, Dar es Salaam, Tanzania; School of Pharmacy, Muhimbili University of Health and Allied Sciences, PO Box 65013, Dar es Salaam, Tanzania; School of Pharmacy, Muhimbili University of Health and Allied Sciences, PO Box 65013, Dar es Salaam, Tanzania; School of Pharmacy, Muhimbili University of Health and Allied Sciences, PO Box 65013, Dar es Salaam, Tanzania; School of Medicine, Muhimbili University of Health and Allied Sciences, PO Box 65001, Dar es Salaam, Tanzania; Liwale District Hospital, PO Box 28, Lindi, Tanzania; School of Medicine, Muhimbili University of Health and Allied Sciences, PO Box 65001, Dar es Salaam, Tanzania; Regional Administrative Secretary, Dar es Salaam Region, PO Box 5429, Dar es Salaam, Tanzania; Pharmacy Council, Ministry of Health, Community Development, Gender, Elderly and Children, PO Box 31818, Dar es Salaam, Tanzania; School of Pharmacy, Muhimbili University of Health and Allied Sciences, PO Box 65013, Dar es Salaam, Tanzania; United Nations Children’s Fund, Bâtiment BIT, 4 Route des Morillons, CH-1211 Geneva 22, Switzerland; United Nations Children’s Fund, 3 United Nations Plaza, New York, NY 10017, USA

## Abstract

**Background:**

In 2017, Tanzania launched the National Action Plan for Antimicrobial Resistance (NAPAR), 2017–2022 and implementation of antibiotic stewardship programmes (ASPs) was one of the agendas. Since the launch of the National Action Plan, no study has been done to assess its implementation.

**Objectives:**

To explore the experiences of prescribers and dispensers on implementing ASPs among paediatric patients attending Regional Referral Hospitals (RRHs) in Tanzania.

**Methods:**

An exploratory qualitative study was conducted among key informants, in 14 RRHs in Tanzania between July and August 2020. A total of 28 key informants, 14 dispensers in charge of pharmacies and 14 medical doctors in charge of paediatric departments (prescribers), were interviewed. A hybrid thematic analysis was conducted on the gathered information.

**Results:**

Most of the study participants were not conversant with the term ‘antibiotic stewardship’. Some had heard about the programmes but were not aware of the activities involved in the programme. Those who were knowledgeable on ASPs mentioned the lack of existence of such programmes in their settings. They further added that absence or limited knowledge of the stewardship concepts may have influenced the current poor practices. Barriers to the implementation of ASPs mentioned were lack of laboratory facilities to support culture and susceptibility tests, lack of materials and reagents, management pressure to prevent loss or to generate income, patients’ influence and limited training opportunities.

**Conclusions:**

Despite launching the NAPAR in 2017, we found limited implementation of ASPs in the management of paediatric patients. This study highlighted some barriers and identified possible intervention points.

## Introduction

Antimicrobial resistance (AMR) has become a public health threat of the contemporary era.^1^ Until recently, most antimicrobials were effective to eliminate the majority of infections.^2^ However, the emergency and acceleration of resistance as a result of irrational antimicrobial use and the microorganism evolution has rendered most of these previously effective regimens ineffective.^3,4^ Globally, it is estimated that 1.27 million deaths occur every year due to AMR.^2^ Urgent countermeasures are necessary to reduce the morbidity and economic strain as a result of long hospital stays and expensive medication, especially for developing countries.^5^

In 2016, the International Health Regulations (IHR) Joint External Evaluation (JEE) highlighted AMR as a major problem in Tanzania, with high levels of misuse of antimicrobials.^6^ In comparison with adults, children have a higher risk of AMR because they are more likely to succumb to infectious disease and hence consume antimicrobials the most.^7^ Current country data on the burden of AMR among children is lacking in Tanzania, but findings from the Global Antimicrobial Resistance Partnership (GARP) surveillance report of 2015 showed an increased trend of co-trimoxazole resistance in *Streptococcus pneumoniae* from 25% to 80% for children under 5 years of age, within the span of 6 years.^8^ Additionally, ESBLs were found in 25%–40% of *Escherichia coli* strains in both community and hospital isolates, whereby, more than 50% of the isolates were from children.^8^ Moreover, data from tertiary health facilities have shown the majority of Gram-negative isolates exhibit resistance to ampicillin and gentamicin, ranging from 37% to 89%. These antibiotics are considered first-line treatment for children.^9–11^ Furthermore, resistance to third-generation cephalosporins, considered to be second-line treatment in Tanzania, ranges from 25% to 58%.^9,10^

Although dispensing of antibiotics in a hospital set-up is controlled by prescription,^12^ antimicrobial misuse is still high. Contributing factors include, among others: polypharmacy;^13^ non-evidence-based prescription;^12^ limited availability of antibiotics in public hospitals,^14^ hence patients access antimicrobials through community pharmacies where control is limited; and lack of health assurance, which can lead to cash purchase of incomplete doses.^15^ Some of these factors are complex and require a systemic solution while other factors can be managed if the prescriber and dispenser implement antimicrobial stewardship in their daily practice. An antimicrobial stewardship programme (ASP) is expected to foster appropriate use of antimicrobials by optimizing antibiotic selection, appropriate dosage and duration of therapy to maximize clinical cure and prevent further emergence of resistance.^16^ At implementation levels, some of these programme strategies include antibiotic use evaluation and monitoring in health facilities, updating the standard treatment guideline, training of healthcare providers (HCPs),^17^ creating community awareness and one health initiatives.^17^

Implementation of antimicrobial stewardship in Tanzania was challenged by unavailability of an approved framework for use in hospital settings.^6^ Following the global commitment to implement ASPs to curb AMR and the recommendation that all acute care hospitals should implement ASPs,^18^ Tanzania launched the National Action Plan on Antimicrobial Resistance (NAPAR) in 2017–22.^17^ This outlined key strategies for a coordinated response and strengthening of the national antimicrobial stewardship programmes. As a result, hospital formulary and other AMR policies have been developed, training of HCPs, including prescribers, pharmacists, nurses and laboratory personnel has been conducted in health facilities and is ongoing, and medicine and therapeutic committees have been established and capacitated, among others.

Therefore, this study reports the experiences of prescribers and dispensers on the implementation of ASPs for the paediatrics population in the selected Regional Referral Hospitals (RRHs) in Tanzania, 3 years after the launch of the NAPAR.

## Methods

### Study design, study site and study population

**Figure 1.**
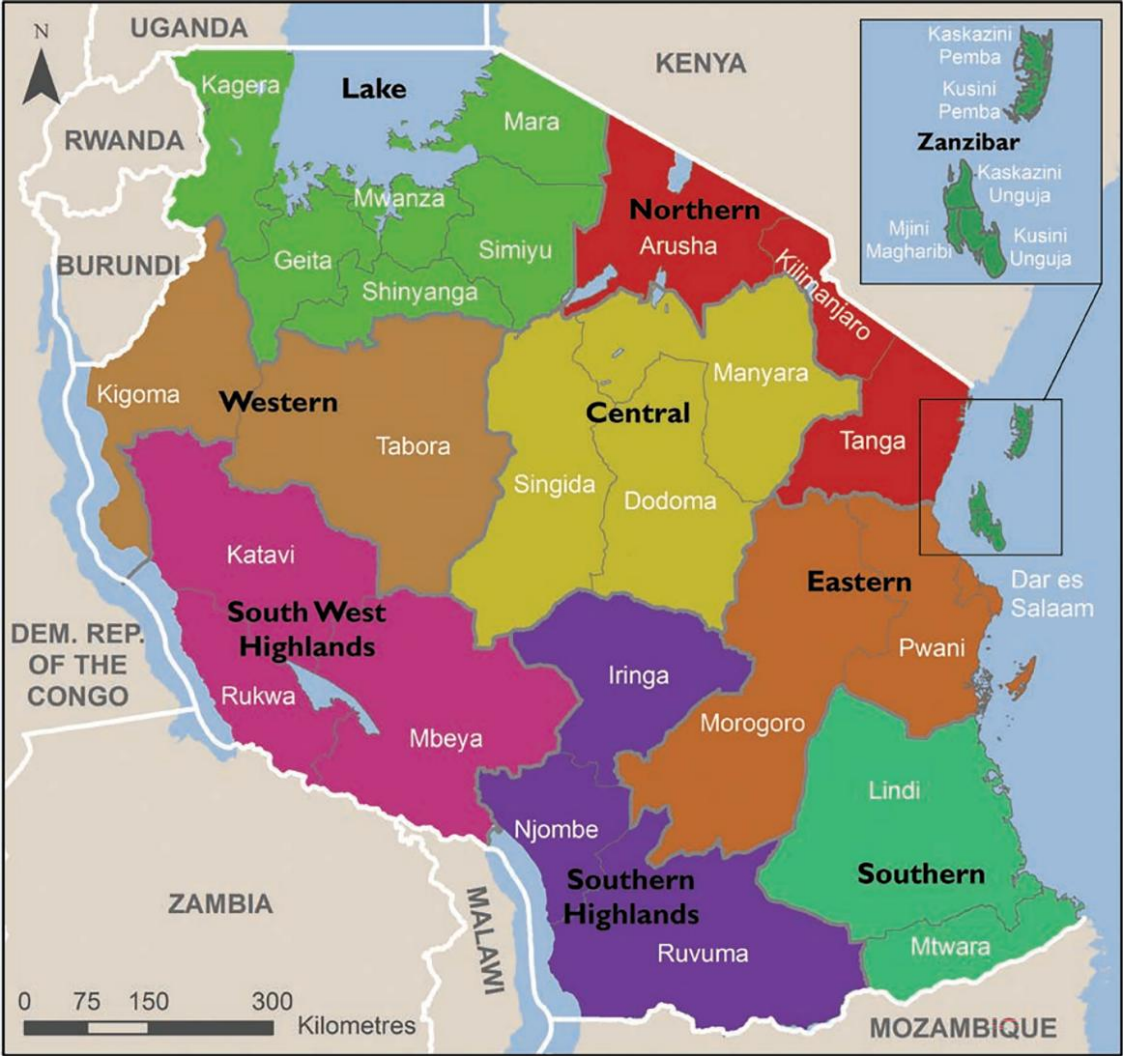
Tanzania’s map indicating study sites (zones) where the RRHs were selected (source: encyclopedia.pub/entry/11084).

Exploratory qualitative research using a semi-structured interview guide was used to explore the experience of prescribers and dispensers on the implementation of ASP in 14 RRHs in Tanzania between July and August 2020. Two RRHs from each zone were selected to represent seven administrative zones in mainland Tanzania. The RRHs were selected from: Southern West zone: Mbeya and Songwe regions; Southern Highlands zone: Njombe and Iringa regions; Lake zone: Mara and Mwanza regions; Central zone: Singida and Dodoma regions; Eastern zone: Pwani and Dar es Salaam regions; Northern zone: Manyara and Kilimanjaro regions; Southern zone: Lindi and Ruvuma regions (Figure 1). The regions were selected based on socioeconomic activities, which are likely to influence antimicrobial use. The RRHs were selected because they were capacitated to implement ASP as a key strategy in fighting the AMR tragedy in the country.

This study involved key informants in charge of the paediatric and pharmaceutical units/departments who were purposefully selected to provide both professional and administrative points of view on ASP implementation within their hospitals. One prescriber and one dispenser were enrolled from each RRH. This totalled 28 key informants from the 14 RRHs.

### Study context

In Tanzania, the RRHs fall in the secondary level of a decentralized healthcare service delivery system that provides both curative and preventive services. The RRHs receive patients referred from low-level healthcare facilities as well as self-referral. At large, these facilities procure medicines from the Medical Store Department (MSD) and vertical programmes. In Tanzania, the RRHs are entitled to store, prescribe and issue antibiotics in the reserved class (such as meropenem, clindamycin and cefepime. In health facilities, antibiotics are mainly prescribed by physicians and dispensed by pharmacists or pharmaceutical assistants/technicians. The RRHs were empowered to facilitate the implementation of ASPs as a key strategy in fighting the AMR tragedy. For this reason, the selection of health facilities considered the level/type of the health facility. The selection of participants was based on their experiences of prescribing/dispensing antibiotics to paediatric populations who are affected the most.

### Data collection and analysis procedures

The interview guide (available as Supplementary data at *JAC* Online) was developed in the English language after a comprehensive literature review in the context of ASPs,^19–21^ and piloted with 10 prescribers and 10 dispensers at Muhimbili National Hospital, who understood the respective professional roles in implementing an ASP in Tanzania. Data obtained were excluded from the findings. Questions on the interview guide enquired about: (i) how they understood the concept and components of ASPs; (ii) what is their overall perception and experience on implementing antimicrobial stewardship?; (iii) what the impact of ASPs on antimicrobial use has been, especially in children; (iv) barriers for implementing ASPs; and (v) what were the ASP learning sources and further recommendations. Twenty-eight in-depth interviews were conducted. Data collection and analysis procedures were conducted as elaborated in our recent publication^22^ in reference to Fereday and Muir-Cochrane.^23^

### Ethics

Ethical approval to conduct this study was obtained from Muhimbili University of Health and Allied Sciences (MUHAS), Senate Research and Publications Committee (reference number: MUHAS-REC-3-2020-109).

## Results

### Participants’ sociodemographic characteristics

The sociodemographic characteristics of the participants are presented in Table 1.

**Table 1.** Participants’ sociodemographic characteristics

Characteristics	Prescribers^a^ (*N* = 14)	Dispensers^b^ (*N* = 14)
Age, *n*, mean (SD): 32.8 (4.7) years		
* *18–25 years	0	1
* *26–34 years	8	9
* *35–44 years	6	4
Sex, *n*		
* *Male	5	10
* *Female	9	4
Highest professional education level, *n*		
* *Postgraduate	5	0
* *Graduate	9	10
* *Diploma	0	4
Experience of working with paediatric patients, *n*, median (range): 4.0 (1–11) years		
* *≤2 years	4	5
* *≥3 years	10	9

a Prescribers included clinical officers/assistant medical officers, medical officers, specialists and consultants.

b Dispensers included pharmaceutical assistants, pharmaceutical technicians, pharmacists.

### Understanding the concept and components of ASPs

Most participants stated that they were not conversant with the meaning of the term stewardship itself while some reported having heard about it but were unaware of a stewardship programme for antimicrobials.

*‘I have never heard of that programme as a programme, but if you tell me antibiotic stewardship, I translate, that at an individual level you are like an ambassador to emphasize the proper use of antimicrobials….’ (Prescriber 18)*

*‘I am not well informed except knowledge on antibiotic resistance, those issues of antibiotic stewardship am not aware at all.’ (Dispenser 17)*

*Probably, you could start by informing me what antibiotic stewardship is because, to me, it is a new word. How can someone practice stewardship? (Dispenser 5)*

Those who happened to understand the concept and the components of ASPs programmes supported that the programmes were crucial in facilitating the appropriate use of antimicrobials, especially in children.

*‘Antibiotic stewardship training brings changes because at the beginning most prescribers used to start treatment by prescribing broad-spectrum antimicrobials…now they understand the consequences of treating children with broad-spectrum antimicrobials instead of beginning with first-line antimicrobials…To me, this is the biggest improvement…’ (Dispenser 1)*

*‘…Antibiotic stewardship training has helped me in decision making…as you may know dispensing is not only about issuing medication but providing instruction to patients on how to use the medication because a slight mistake may harm the patients…’ (Dispenser 17)*

Additionally, participants mentioned that collective efforts involving all key stakeholders, including all health professional cadres, is a key way to achieve ASP objectives. They stated that if clinicians, nurses, pharmacists, laboratory personnel and social workers play their roles at the health facilities, antimicrobial stewardship is likely to be implementable.

*‘I think we should all be responsible for implementing antibiotic stewardship from pharmacists, laboratory personnel, nurses, doctors, and the management especially the management …’ (Prescriber 2)*

*‘I think all people should be involved, including dispensers and prescribers. Because without knowledge on antimicrobial stewardship, when a doctor prescribes a wrong antibiotic no one will be able to correct it.’ (Dispenser 23)*

### Barriers towards implementing aspects of ASPs

#### Influence from the hospital management

Participants stated that management at the health facilities contribute to poor implementation of ASPs. They stated that management pressure to prescribe based on the available/near expiry stock were mentioned as facility-level contributors towards poor ASP implementation.

*‘Sometimes when certain antimicrobials are out of stock…example amoxicillin/clavulanic acid, we get calls from pharmacy that only certain antimicrobials are available…so whoever comes we should prescribe what is available,…just like that’ (Prescriber 2)*

‘*In some cases the hospital management instructs us to dispense strong antimicrobials without any supporting evidence…if you resist you will be blamed for contributing to the expiry of expensive antimicrobials, which is a loss to the hospital…’ (Dispenser 23)*

*‘…when antimicrobials are about to expire, prescribers are instructed by the hospital management to prescribe them to patients even when there is no clear indication…it has happened mostly with expensive antimicrobials, for example meropenem…’ (Prescriber 4)*

#### Lack of facilities that support laboratory investigations

Most of the respondents stated the absence of supporting infrastructure such as a well-functioning microbiology laboratory for culture and susceptibility tests in the RRHs. Furthermore, the respondents stated that in cases where the laboratory is available there are usually no reagents for tests. Other participants mentioned that their referral hospitals had never performed culture and susceptibility testing; when required they refer patients to zonal referral hospitals.

*‘…. we treat our patients empirically, we do it by following the treatment guideline, if all drugs fail, culture and sensitivity is needed, we refer the patient to a zonal referral regional hospital…we don’t have a laboratory that can perform culture and sensitivity testing’. (Prescriber 24)*

*‘…in most cases when we order culture and sensitivity tests we do not get timely results, when you try to make a close follow-up, the laboratory personnel will inform you we do not have reagents.’ (Prescriber 10)*

#### Patients’ contribution to poor antibiotic stewardship

Participants viewed patients as contributors to poor antibiotic stewardship. They stated that some patients believe in certain antimicrobials and even when they appear at a health facility, they will influence the prescribers or dispensers to give them the antimicrobials of their choice. On the other hand they stated that some patients do not attend health facilities in fear of costs and thus opt to acquire antimicrobials over the counter without sufficient investigation.

*‘…Some patients come to the facility and insist for a particular antibiotic and when you suggest a different one in line to what they are suffering from they refuse…’ (Prescriber 11)*.

‘*…probably because of financial constraints, some patients come with past prescription of certain antimicrobials…and sometimes request incomplete doses…’ (Dispenser 11)*

‘*….sometimes patients come with prescriptions of certain antimicrobials and request to get half or a quarter of the dosage, for example, I need a 3 days’ dosage…I will use it and return when I get some more money’. (Prescriber 11)*

#### Existence of selective continued medical education (CME) opportunities

Participants stated that most of the CME opportunities were selective in a sense that they only targeted certain diseases or certain groups of healthcare workers. This limited most other cadres on acquiring new knowledge about antimicrobials.

*‘Training on antibiotic stewardship do come, but these opportunities come with specific names or positions of the required participants, and they are usually the same people’ (Prescriber 10)*.

*‘Most of the offered training are on HIV and malaria, I have not heard of training on antimicrobial resistance or anything on stewardship (Prescriber 23)*

#### Limited opportunities for training on antibiotic stewardship

Most of the participants stated absence of opportunities for training on antimicrobial stewardship in their workplaces or a nearby place. They stated that this influenced how they prescribed antimicrobials as they still rely on what they learnt from their college or colleagues.

*‘Personally, I have never received any training since I joined this department…I only use the knowledge acquired from the college/university training…’ (Prescriber 24)*

*‘I have not received any training opportunity since I exited school…’ (Prescriber 2)*

*‘… I learnt antibiotic stewardship issues as a topic during pharmacology sessions in class…’ (Prescriber 23)*

### Sources of information/training on ASPs

Participants who were conversant with ASPs mentioned their sources of knowledge on ASPs to be CME opportunities and workplace colleague learning.

#### CME

Some participants in some sites stated the existence of CME opportunities for providing knowledge on ASPs. They mentioned these CME opportunities to include seminars, workshops, research meetings and online courses. They further stated that awareness of the existence of these opportunities is vital for improving the fight against AMR.

*‘Yes, I attended a certain training on neonatal sepsis in which I learnt a little on antibiotic stewardship, the training helped me a lot on understanding issues associated with antibiotic resistance’. (Prescriber 11)*

*‘Currently we have been instructed not to use a Widal test in diagnosing typhoid, instead conduct stool culture and the message has been communicated through the departmental presentation…if someone was not present during presentation they may continue with the old practice…What we do is to approach him/her individually and explain on what transpired in that presentation…’ (Prescriber 10)*

### Recommendations

Participants recommended several measures to strengthen the implementation of hospital ASPs.

#### Engaging the community in the implementation of ASPs

Participants stated that in order to implement ASPs and hence fight antibiotic resistance, provision of education on appropriate use of antimicrobials, encouraging hospital-seeking behaviour and proper disposal of expired medication is important.

*‘I think the community need to be involved in the stewardship programme, the same as what has been done in the COVID-19 fight, through different communication media, we need to insist the community on the rational use of antimicrobials and advocate hospital-seeking behaviour, in order to fight antibiotic resistance…’ (Dispenser 2)*

*‘Also we must continue to provide education to the community on proper ways of disposing antimicrobials…If a patient has expired drugs at home they should not dispose them to the surroundings, it is not a proper way and it may lead to antibiotic resistance…’(Dispenser 4)*

#### CME on antibiotic stewardship for healthcare workers

Furthermore, participants recommended conducting CME for healthcare workers. This includes continued emphasis on prescriptions based on culture and susceptibility tests.

*‘On the continuous medical education on stewardship, emphasis on culture and sensitivity is important since it plays a key role in reducing the burden of antibiotic resistance. As it helps in isolating the bacteria responsible for a particular medical condition before prescription of antimicrobials…’ (Dispenser 3)*

#### Health facilities should establish antibiotic stewardship committees

Participants stated that health facilities should establish and build capacity of a stewardship committee that will oversee the programme activities within the facility. In some set-ups, these teams do exist but are not functional.

*‘I think in order to fight antibiotic resistance, hospital management should make efforts to establish and strengthen antibiotic stewardship committees. For example in our setting, some people received letters of appointment as members of stewardship programmes but nothing has been done to date by the committee…We expected this committee to bring changes in our setting through provision of education because antibiotic resistance is a very sensitive issue, but so far nothing has been done’. (Dispenser 16)*

## Discussion

We conducted this study to evaluate the implementation of ASPs following the launch of NAPAR in 2017.^17^ The study found most of the participants were unaware of ASPs, most of the prescribers and dispensers stated that they were not conversant with the meaning of the term stewardship itself and others were not aware of what it entails. Those who were knowledgeable on ASPs mentioned the lack of existence of such programmes in their settings. They further added that absence or limited knowledge on the stewardship concepts may have influenced the current poor practices. Barriers to the implementation of ASPs were mentioned to be the lack of laboratory facilities and/or laboratory reagents, pressure from the hospital management, limited training opportunities and the patients themselves.

It is alarming to find healthcare workers in referral hospitals unaware of ASP, 3 years post the launch of NAPAR in Tanzania. These findings relate to a recent study conducted in district healthcare facilities, 1 year after the launch of the national action plan, where only one-third were implementing ASPs despite all of them being aware of the state of AMR in Tanzania.^16^ Similarly, Hall *et al.*^24^ reported the majority (71%) of physicians being unfamiliar with the term ‘antimicrobial stewardship’ although almost all (91%) were aware that AMR is becoming a public health problem in Tanzania. Evidence suggests dedicated implementation of ASP activities can optimize the use of antimicrobials and reduce AMR,^25^ although in most African countries, the common impeding factors to implementation remain to be limited financial capacity and infrastructure to fully execute.^26^ Keeping all factors in perspective, the NAPAR began execution 1  year (2017) after Tanzania scored the lowest on the WHO AMR indicator, where the country was rated with no capacity to implement ASP because of lack of an approved framework for use.^6^ The NAPAR clearly states how ASP will be implemented and evaluated at different levels of health facilities in Tanzania,^17^ but considering the starting position of the country, the 2017–22 time frame may have been a shorter time to establish frameworks and implement. Moreover, the modality of implementation and progress was not made public as it would have informed our study site selections.^27^

At the RRH level, several factors were reported to impede the implementation of hospital ASPs. This included lack of laboratory facilities to support culture and susceptibility and lack of materials and reagents to facilitate testing. Although the Tanzanian standard treatment guidelines dictate treatment to be backed up by laboratory evidence,^28^ this is deterred by limited investment in the laboratory infrastructure in most of the hospital settings.^29^ Correct identification of pathogens and susceptibility testing is complex and capital intensive^30^ and financial constraints facing the health system in Tanzania and the majority of African countries have normalized empirical treatment because it is relatively easy and inexpensive, although it may further increase the burden of AMR.^31^ Sometimes it is the perception of the prescribers themselves who argue on urgency of treatment, high patient load and limited time to attend patients, and hence cannot wait for culture and susceptibility results for all patients.^31,32^ This practice is expected to be annihilated by the practical implementation of ASP in health facilities as per the NAPAR.

Pressure from the hospital management to increase financial income or, in some circumstances, prevent loss by prescribing antimicrobials close to their expiry date, regardless of the illness, as well as limiting prescription to what is available in stock was also named as one of the barriers for implementing ASPs. This mainly eliminates room for evidence-based treatment and hence exacerbates the burden of AMR. It is known that successful implementation of stewardship activities requires unwavering commitment from senior leadership,^33^ to ensure evidence-based treatment, proper planning to avoid expiry of medications, accessibility of the required stock of antimicrobials, establishment of hospital therapeutic committees and conducting of regular audits of antibiotic use, all of which are requirements of ASP activities as per NAPAR.^17^ Like other studies,^22,34^ findings from this study support the notion that implementation of an ASP without the commitment of senior leadership is likely to fail.

Another observed barrier to implementation of ASPs in health facilities is the patients themselves. Patients are observed to drive poor implementation of ASPs by influencing the healthcare workers not to align to standard patient management practice and instead act as they are requested.^35,36^ Patient pressure is reported to influence more than half of a clinicians’ prescriptions where they find it hard to refuse to children, the elderly and people they generally like.^36^ Moreover, rigid patient attitudes and financial constraints were also reported as other patient-related barriers to implementing ASPs. Being financially challenged adds a layer of complexity towards implementation of ASPs;^37^ poverty is known to contribute to AMR because the clinician may be forced to prescribe based on clinical presentation because the patient cannot afford to pay for the necessary laboratory investigation.^38^ Moreover, the patient may opt for a different antibiotic because the one prescribed is expensive.^39^ The growing burden of AMR is expected to continue to strain the limited resources with the escalating treatment cost as a result of morbidity and unavoidable long-hospital stays for treatment,^40^ if no measures are taken to halt its progression. The patients’ rigid attitude regarding not conforming to the instructions provided by the healthcare workers is also reported elsewhere, and mass education is observed to reduce these practices.^22,38^

Lastly, one of the activities to be performed in ASPs is training and capacity building of the healthcare workers, and according to the NAPAR this has been an ongoing activity since its launch in 2017. However, findings from the current study have shown limited training opportunities, in terms of who can access this training and selective CME, where most do not focus on ASP. This is reported as a major contributor to having limited understanding of ASPs. Education is an important strategy for adoption and maintenance of ASPs. For public hospitals in Tanzania, most of these are either government led, or donor initiated, which may not be sustainable in the long term.^26,39^ In the context of limited resources, utilization of existing collective skills and knowledge in the multidisciplinary teams, as well as accessing different applicable educational resources available through our different professional associations is critical to ensure sustainability of stewardship practices.^41^

In line with these efforts, regular monitoring of compliance following education is critical to point out gaps and areas of intervention to ensure adequate implementation of ASPs in RRHs. Moreover, unrestricted access to antimicrobials in community pharmacies may water down ASP efforts in hospital set-ups. Hence enforcement of the Food, Drug and Cosmetic Act of 2015, which prohibits sale or administration of antibiotics without prescription,^42^ is necessary.

### Study limitations

This study did not involve other healthcare cadres such as nurses and medical laboratory personnel. However, prescribers and dispensers are indicated as core implementers of ASPs.^43^

### Conclusions

Despite the launch of the National Action Plan on Antimicrobial Resistance in 2017, we found limited implementation of ASPs, especially for paediatric patients in the RRHs in mainland Tanzania. We therefore recommend CME on ASPs for healthcare workers and AMR awareness in the community so as to promote rational use of antimicrobials in the paediatric population. Furthermore, since the implementation of ASPs is multifaceted, requiring the involvement of all stakeholders, i.e. policymakers, regulatory authorities, funders, healthcare providers and the community, all hospital settings should establish and empower these committees to oversee the implementation of ASPs.

## Supplementary Material

dlac118_Supplementary_Data

## Supplementary data

The interview guide is available as Supplementary data at *JAC* Online.
