# Supplementary material for: Implementation of antibiotic stewardship programmes in paediatric patients in regional referral hospitals in Tanzania: experience from prescribers and dispensers
Source: JAC Antimicrob Resist. 2022 Nov 23;4(6):dlac118. doi: 10.1093/jacamr/dlac118 (PMC9683393; doi:10.1093/jacamr/dlac118)
Supplement: dlac118_Supplementary_Data [file dlac118_supplementary_data.doc]

**ENGLISH FORMAT**

**KEY INFORMANT INTERVIEW GUIDE (PRESCRIBER/DISPENSER)**

**REGION.....................................**

**INTRODUCTION**

Before I begin, I would like to thank you for taking this time to participate in this study. The information you provide will help us to better understand your experience, perception or opinions about antibiotic resistance among prescribers and dispensers in Tanzania. Your responses will be kept confidential. There is no right or wrong answer and the intent of the interview is to understand your experience and perceptions/opinions. You can feel free to be open and honest as you can but if there is any question you would prefer not to answer or if you would like to stop the interview, please let me know. There will be no consequences if you decide not to answer any question (s) or if you would like to stop the interview.

**PARTICIPANT DEMOGRAPHICS** **(*Fill before switching on the audio recorder*)**

**Name of Interviewer(s)** (use initials, i.e. GMB)__________________

**Date of interview** (dd/mm/yr)__________________

**Participant number** (i.e. Mwanza 01, Dar 01) ________

**Age** __________yrs

**Gender**_________________

**Name of the hospital**___________________

**Cadre** (Prescriber/ Dispenser)_________________________

**Highest professional education level** (tick the appropriate)

( ) Certificate

( ) Diploma

( ) Graduate

( ) Postgraduate

**How long have you worked in paediatric unit/ paediatric patients** ________________yrs

**INTERVIEW QUESTIONS *REMINDER: PLEASE SWITCH-ON THE AUDIO RECORDER***

1. Antibiotic resistance is currently considered a public health problem. Kindly tell us what do you know about this problem. Probe on:

- Meaning of Antibiotic resistance
- How does antibiotic resistance occur?
- Activities(factors) contributing to increased antibiotic resistance
- What efforts are done or have been done by the hospital management, staff and users to address the problem of antibiotic resistance.

Probe;

- - Prescribers
  - Dispensers
  - Patients
  - Community
  - Policy makers etc

1. Thank you very much for detailed responses, coming to your own side as a dispenser/prescriber, we understand that it is crucial that you receive regular refresher’s training. What opportunities/training have you got on Antibiotic resistance? Probe on;

- How frequent are the opportunities?
- Opportunities in the last one year
- Opportunities in the last five years
- How easy is it to get the opportunities?
- Contents/composition of each training
- Benefits of the training
  - In improving delivery
  - In behavior change
  - In improving skills

1. To what extent do you know about antibiotic stewardship program?

Probe:

- Meaning of antibiotic stewardship
- Responsible persons for antibiotics stewardship
- Activities performed under the umbrella of antibiotics stewardship
- How have you been involved in the implementation of antibiotic stewardship activities

1. What is your overall perception or experience about the antibiotic stewardship? Probe: How antibiotic stewardship have impacted the quality of delivery of antibiotics? have you noticed changes in delivery of antibiotics? How have they changed?
2. What do you think is need to continue improving the antibiotic stewardship activities? Probe: policies/guidelines, staffing, support from ministry, supervision, mentorship
3. What would you like to recommend on antibiotics stewardship program? Probe: What is needed to ensure that antibiotic stewardship activities continues to be done to address the antibiotic resistance?

Is there anything else you would like to say about antibiotic stewardship? Probe: challenges, budget etc

Thank you for your time!!!!

***REMINDER: PLEASE SWITCH-OFF THE AUDIO RECORDER***
